# Attenuation of Novelty-Induced Hyperactivity of *Gria1-/-* Mice by Cannabidiol and Hippocampal Inhibitory Chemogenetics

**DOI:** 10.3389/fphar.2019.00309

**Published:** 2019-03-29

**Authors:** Teemu Aitta-aho, Milica Maksimovic, Kristiina Dahl, Rolf Sprengel, Esa R. Korpi

**Affiliations:** ^1^Department of Pharmacology, Faculty of Medicine, University of Helsinki, Helsinki, Finland; ^2^Research Group of the Max Planck Institute for Medical Research at the Institute of Anatomy and Cell Biology, Heidelberg University, Heidelberg, Germany

**Keywords:** AMPA receptors, cannabidiol, DREADD, hippocampus, c-Fos, hyperactivity, novelty, hM4Gi

## Abstract

Gene-targeted mice with deficient AMPA receptor GluA1 subunits (*Gria1-/-* mice) show robust hyperlocomotion in a novel environment, suggesting them to constitute a model for hyperactivity disorders such as mania, schizophrenia and attention deficit hyperactivity disorder. This behavioral alteration has been associated with increased neuronal activation in the hippocampus, and it can be attenuated by chronic treatment with antimanic drugs, such as lithium, valproic acid, and lamotrigine. Now we found that systemic cannabidiol strongly blunted the hyperactivity and the hippocampal c-Fos expression of the *Gria1-/-* mice, while not affecting the wild-type littermate controls. Acute bilateral intra-dorsal hippocampal infusion of cannabidiol partially blocked the hyperactivity of the *Gria1-/-* mice, but had no effect on wild-types. The activation of the inhibitory DREADD receptor hM4Gi in the dorsal hippocampus by clozapine-*N*-oxide robustly inhibited the hyperactivity of the *Gria1-/-* mice, but had no effect on the locomotion of wild-type mice. Our results show that enhanced neuronal excitability in the hippocampus is associated with pronounced novelty-induced hyperactivity of GluA1 subunit-deficient mice. When this enhanced response of hippocampal neurons to novel stimuli is specifically reduced in the hippocampus by pharmacological treatment or by chemogenetic inhibition, *Gria1-/-* mice recover from behavioral hyperactivity, suggesting a hippocampal dysfunction in hyperactive behaviors that can be treated with cannabidiol.

## Introduction

Neurological and psychiatric brain diseases cause vast harm and excessive costs to affected individuals and the society at large (DiLuca and Olesen, 2014). There are many clinical evidence-based pharmacological and non-pharmacological treatment options (Millan et al., 2015a,b), but their efficacy should be greatly improved. Unfortunately, there has been a slow progress in neuropsychiatric drug development, especially for schizophrenia, schizoaffective disorder and bipolar disorder, justifying further efforts in finding testable mechanisms, drug candidates and novel targets (Vazquez et al., 2017).

Animal models play an important role in testing mechanisms how neuronal modulations and drug effects are mediated into behavioral responses (Cryan and Slattery, 2007; Forrest et al., 2014), even if they cannot reproduce a full repertoire of symptoms in often heterogeneous brain diseases. Especially difficult are the models of psychosis, since in animals the behavioral outcomes are usually related to motor functions as they are most easily quantifiable. In addition to lithium therapy, presently there are no specific drugs for the treatment of bipolar disorder with mania (Millan et al., 2015b). A simple symptom in that illness is the mania-like hyperactivity, which can be habituated in a constant environment. A mouse line with deficient AMPA-type glutamate receptor GluA1 subunits [the *Gria1-/-* mouse line (Zamanillo et al., 1999)] has been proposed as an animal model for hyperactive disorders, including schizoaffective disorder and bipolar disorder with mania (Fitzgerald et al., 2010; Barkus et al., 2011; Procaccini et al., 2011). This is based on (1) robust novelty-induced hyperactivity that is eventually habituated, leading to unaltered diurnal home-cage activity in the *Gria1-/-* mice (Vekovischeva et al., 2001; Fitzgerald et al., 2010; Sanderson et al., 2010; Procaccini et al., 2011), (2) consistent attenuation of this behavior by chronic treatment with drugs having antimanic efficacy in patients (Maksimovic et al., 2014a,b), and (3) linkage equilibrium of *GRIA1-*gene polymorphisms in psychotic disorders (Ripke et al., 2013, 2014; Devor et al., 2017).

The basic characteristics of *Gria1-/-* mice do not deviate from *Gria1+/+* wild-type littermates (WT). Physical health, body weight, food consumption, nociception, neurological, motor, sexual, sensory functions, and circadian rhythm appear normal (Vekovischeva et al., 2001, 2004; Bannerman et al., 2004; Hartmann et al., 2004; Feyder et al., 2007; Chourbaji et al., 2008; Fitzgerald et al., 2010; Procaccini et al., 2011). Extensive behavioral comparison of *Gria1-/-* and *Gria1+/+* mice suggests a schizophrenia- and depressive-like phenotype. Deficiency in prepulse inhibition of an acoustic startle reflex is indicative of psychosis-related properties (Wiedholz et al., 2008) and increased learned helplessness as deficits in coping skills in aversive situations indicative of depressive phenotype of *Gria1-/-* mice (Chourbaji et al., 2008). Short term spatial working memory of *Gria1-/-* mice is disrupted (Reisel et al., 2002; Sanderson et al., 2009). However, despite the prominent role of GluA1-type AMPA receptors in hippocampal synaptic plasticity *Gria1-/-* mice can form spatial reference memory (Zamanillo et al., 1999).

The most striking and reproducible behavioral response of *Gria1-/-* mice has been hyperactivity provoked by a novel environment. They have normal locomotion compared to their WT littermates in a familiar home-cage environment (Wiedholz et al., 2008; Procaccini et al., 2011), but when transferred to novel environment, they double their locomotor activity (Vekovischeva et al., 2001; Bannerman et al., 2004; Chourbaji et al., 2008; Fitzgerald et al., 2010; Procaccini et al., 2011) and fail to habituate (Barkus et al., 2011; Sanderson and Bannerman, 2012).

This increased activity is sustained, and it takes 30 to 40 min before the *Gria1-/-* mice show signs of habituation to the novelty. Thus, any confrontation with a novel signal, such as a new object or littermate, induce an aberrant reaction in *Gria1-/-* mice due to the lack of habituation (Wiedholz et al., 2008). Similarly, in sociability and resident-intruder tests *Gria1-/-* mice show remarkably low level of intermale aggression, suggesting poor ability to adapt to social encounters (Vekovischeva et al., 2004).

We have recently found that *Gria1-/-* mice react to novel environment by increased neuronal activation of the hippocampi, as shown by the enhanced immediate early gene expression (Procaccini et al., 2011). In the present study, we used *Gria1-/-* mice to analyze the effect of systemic application and acute hippocampal infusion of the non-psychoactive phytocannabinoid cannabidiol (CBD) (Izzo et al., 2009) on the hyperactivity and hippocampal c-Fos expression in *Gria1-/-* and controls. CBD has been shown potential as treatment for schizophrenia (Schubart et al., 2014). We also tested whether selective inhibition of the dorsal hippocampal principal neurons is sufficient to down-regulate the hyperactivity by using the activation of the virus-mediated, cell-type specific expression of an inhibitory designer receptors exclusively activated by designer drug (DREADD) (Roth, 2016), the hM4Gi receptor (Armbruster et al., 2007).

In our experiments, both the CBD and DREADD approaches attenuated the behavioral response to novelty of *Gria1-/-* mice, indicating that the paradoxical hippocampal activation is associated with hyperactivity of this mouse model. Importantly, acute infusion of CBD specifically into the hippocampus reduced the abnormal hyperactivity of the *Gria1-/-* knockout mice but did not affect the spontaneous activity of the wild-type littermates.

## Materials and Methods

### Animals

*Gria1-/-* mice (*Gria1^-/-^*, *Gria1*^tm1Rsp^; MGI:2178057) and their *Gria1+/+* wild-type littermate controls (WT) were from heterozygous breeding, generated by inactivation of the *Gria1* gene (Zamanillo et al., 1999) and genotyped as described (Vekovischeva et al., 2001). The *Gria1-/-* mouse line is available at the Jackson Laboratory (B6N.129-*Gria1*^tm1Rsp^/J, stock number: 019011). Mice were group-housed under standard laboratory conditions (12-h light-dark cycle; lights on at 6:00 A.M.; temperature 20–23°C; relative humidity 50–60%; aspen chip beddings). For locomotor activity, a total of 24 *Gria1-/-* (14.12 ± 0.92 weeks; 24.58 ± 1.03 g) and 23 WT mice (11.14 ± 0.66 weeks; 24.63 ± 0.92 g) were used.

All experimental procedures were approved by the State Provincial Government of Southern Finland (ESAVI-0010026/041003/2010). All efforts were made to minimize the number and suffering of animals.

### Novelty-Induced Locomotor Activity

Locomotor activity in a novel environment after acute systemic cannabidiol treatments was observed in plastic cages (40 cm × 30 cm × 20 cm) as described in detail (Procaccini et al., 2011; Maksimovic et al., 2014b). At least 1 h before the tests was allowed for the animals to habituate to the experimental room. Horizontal movements of eight mice, placed in visually isolated cages in a sound-attenuated room at the light intensity of 175 lx, were simultaneously recorded for 2 h using EthoVision Color-Pro 3.0 video tracking software (Noldus Information Technology, Wageningen, Netherlands). The same method, but only during 30-min recordings, was used to assess the effects of intrahippocampal CBD infusions and chemogenetic inhibition of hippocampal neurons (see below).

### c-Fos Immunostaining

The animals were quickly decapitated after 2-h novelty-exploration, the brains dissected, frozen on dry ice and stored at -80°C. Fourteen-μm thick sections were cut on a cryostat (Leica CM 3050 S; Leica Microsystem, Nußloch, Germany), thaw-mounted on Fisher Superfrost Plus slides (Menzel-Glaeser, Braunschweig, Germany) and stored at -80°C. To obtain sections of the ventral hippocampi, we changed the plane of cutting to horizontal one after we had collected the coronal sections until Bregma -2.4 mm (Franklin and Paxinos, 2008) as described earlier (Procaccini et al., 2013; Maksimovic et al., 2014b).

In immunohistochemistry, the protocol described in Procaccini et al. (2011) was followed. In brief, the sections were thawed, air-dried, marked with hydrophobic pen (Daido Sangyo, Tokyo, Japan) and all incubations performed using so-called liquid bubble technique. The sections were fixed with ice-cold 4% paraformaldehyde in Tris-buffered saline (TBS; in mM: Tris, 50; NaCl, 150; pH 7.4) for 10 min. As a washing medium, we used TBS supplemented with 0.05% Tween 20 (TBST) between incubations. Endogenous peroxidases were blocked by 0.3% H_2_O_2_ in methanol. Endogenous proteins were blocked with 10% normal horse serum (Sigma-Aldrich) and avidin blocking solution (Avidin/Biotin blocking kit; Vector Laboratories, Burlingame, CA, United States) diluted in TBST containing 1% bovine serum albumin (BSA; Sigma-Aldrich). Sections were incubated with goat anti-c-Fos antibody (1:1000; Santa Cruz Biotechnology, Santa Cruz, CA, United States) in TBST/1% BSA and biotin blocking solution (Avidin/Biotin blocking kit) overnight at 4°C, followed by 30 min in biotinylated horse anti-goat secondary antibody (1:200; Vector Laboratories). Avidin–biotin peroxidase complex (Vectastain Standard Elite; Vector Laboratories) and diaminobenzidine with nickel sulfate intensification (DAB Substrate kit; Vector Laboratories) were used for visualization. Then, the sections were dehydrated in gradual ethanol solutions (70, 96, and 99.5%), rinsed in Histoclear (National Diagnostic, Atlanta, GA, United States) and finally coverslipped with DPX mounting medium (BHD Chemicals, Poole, United Kingdom).

In quantifying the c-Fos-positive cells, photomicrographs from anatomically-matched sections were captured using a light microscope with a 10× objective (Leica DMR, Leica Microsystems, Wetzlar, Germany) and a CCD camera (Leica DC 300). The analysis was carried out blind to the treatment and genotype. Detection of c-Fos-positive cells was automatic, using ImageJ software (National Institutes of Health, Bethesda, MD, United States), by setting a constant threshold and ‘region of interest’ area based on brain atlas (Franklin and Paxinos, 2008). Analysis comprised bilaterally-obtained values from one or two sections (for dorsal and ventral hippocampi) of 12 brain areas (as shown in Figure 2 and Supplementary Table S1). Apart from the hippocampus, expression of c-Fos protein was studied in those regions that may have a role in explorative locomotor task or where relatively high c-Fos expression is observed (Montag-Sallaz et al., 1999).

### Intrahippocampal Injections of CBD

The hippocampus was targeted with CBD injections by microinjection cannulae. Mice were anesthetized with isoflurane (induction 5%, 2% maintenance; Vetflurane, Virbac, Carros, France), and implanted with bilateral 26-gauge stainless steel guide cannulae (Plastics One, Roanoke, VA, United States) into the dorsal dentate gyrus using a stereotactic device (David Kopf Instruments, Tujunga, CA, United States). Dental cement (Simplex Rapid, Associated Dental Products, Ltd., Swindon, United Kingdom) and two stainless steel screws were used to secure the cannula placement. The coordinates used were 1.9 mm posterior to bregma, 1.0 mm lateral to midline, and 1.0 mm ventral to skull level. Stainless steel obturators were inserted to keep cannulae unobstructed during the 1-week recovery period. Animals’ recovery was monitored daily and none showed signs of distress. Prior to experiments, the mice were gently handled and CBD was injected through the guide cannulae by using 31-gauge internal stainless-steel injectors protruding an additional 1.0 mm below the guide cannulae. The injectors were connected to a microsyringe (Hamilton, Bonaduz, Switzerland) via polyethylene tubing. CBD (5 μg/side) or vehicle was injected in a volume of 0.2 μL at 0.1 μL/min by an infusion pump (kDScientific, Holliston, MA, United States). After the injection, the injectors remained in place for 1 min to allow solutions to diffuse into the tissue. The measurement of novelty-induced locomotor activity was recorded immediately after the injections as described above. After the experiments, the injector tip placements were confirmed histologically.

### Chemogenetics

Dorsal hippocampal dentate gyrus was stereotactically targeted in isoflurane anesthesia as described above for CBD infusions. To each mouse, a total volume of 0.15 μL of AAV5-CaMKIIα-hM4Gi-mCherry viral vector (3.4 × 10^12^ genome copies/mL, University of North Carolina, Gene Therapy Center, Chapel Hill, NC, United States) was injected through a 31-gauge injection cannula using a microsyringe and an injection pump as described above. The animals recovered for 3 weeks. Thirty minutes prior to behavioral experiments the animals were administered clozapine-*N*-oxide (CNO) at 3 mg/kg i.p. in home cage. Non-DREADD-transduced mice acted as controls to test for DREADD receptor-non-specific effects of CNO. The measurement of novelty-induced locomotor activity was recorded immediately after the injections. After the experiments, the animals were deeply anesthetized with pentobarbital (Mebunat, Orion Pharma, Espoo, Finland), transcardially perfused with 0.1 M phosphate-buffered saline (PBS) followed by 4% paraformaldehyde in PBS. Brains were removed, post-fixed overnight at 4°C, and cryoprotected in 30% sucrose in PBS at 4°C until sunk. Forty-μm-thick coronal sections were cut by cryostat (Leica CM 3050 S; Leica Microsystem, Nußloch, Germany). To visualize the DREADD receptors, mCherry tag immunohistochemistry was performed. In brief, brain sections were washed at RT in 0.1 M PBS, blocked with 1% BSA (Merck, Darmstadt, Germany) supplemented with 0.3% Triton X-100 (Fisher Scientific, Fair Lawn, NJ, United States) in 0.1 M PBS. Sections were then incubated overnight at RT in primary antibody (rabbit anti-mCherry, 1:800, ab167453, Abcam, Cambridge, United Kingdom) diluted in blocking buffer, washed in PBS, incubated in secondary antibody (donkey anti-rabbit AlexaFluor 594, 1:1000, ab150076, Abcam) for 2 h at RT, washed in PBS, and mounted on microscope slides and coverslipped. Digital images were captured with a Zeiss Axioimager Z1 microscope using Apotome optical sectioning (Zeiss, Oberkochen, Germany) and Hamamatsu Orca R2 CCD camera (Hamamatsu, Japan).

### Drugs

The doses of cannabidiol (CBD, from THC Pharm GmbH, Frankfurt, Germany) were based on literature (Zuardi et al., 1991; Moreira and Guimaraes, 2005; Long et al., 2006, 2010, 2012; Gururajan et al., 2011, 2012) and on preliminary dose–response experiments. The drug was dissolved in a mixture of ethanol:Tween 80:saline (1:1:18, which was used as a vehicle for controls) (Todd and Arnold, 2016) and injected (i.p.) in a volume of 10 ml/kg. For intrahippocampal injection, CBD was dissolved in 1% Tween 80 and 10% DMSO in sterile saline. Clozapine-*N*-oxide (CNO, Sequoia Research Products, Ltd., Pangbourne, United Kingdom) was dissolved in 0.5% DMSO in saline.

### Statistical Analyses

Data are expressed as means ± standard errors of the mean (SEM). Statistical analyses were carried out using IBM SPSS Statistics 21 software (IBM SPSS, Inc., Somers, NY, United States) using (repeated measures) two-way ANOVA (genotype, treatment) followed by a Bonferroni *post hoc* test. Statistical significance was set at *P <* 0.05.

## Results

### Effect of Acute Systemic Cannabidiol (CBD) Treatment on Novelty-Induced Locomotor Activity

**FIGURE 1:**
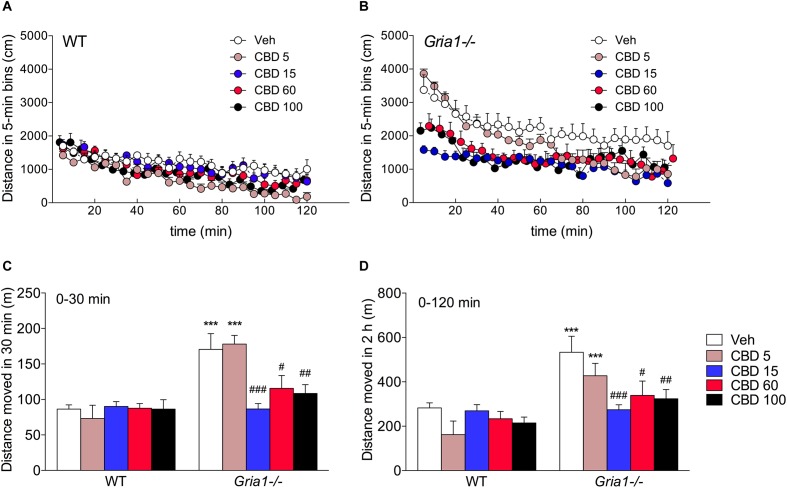
Dose–response analysis of cannabidiol on novelty-induced hyperlocomotion of *Gria1-/-* mice. **(A,B)** Distance traveled in a novel environment in 5-min periods after treatment with vehicle and doses of cannabidiol (mg/kg, i.p.). **(C)** Distance traveled from the beginning of the trial until 30 min. **(D)** Total distance traveled during the whole 2 h test trial. Means ± SEMs are shown for 5–6 mice per group. ^∗∗∗^*P* < 0.001 for the significances of the differences between WT and *Gria1-/-* mice after vehicle treatment; ^#^*P* < 0.05, ^##^*P* < 0.01, and ^###^*P* < 0.001 between vehicle and CBD within the same genotype (Bonferroni post-test). Veh, vehicle; CBD, cannabidiol.

In keeping with our previous results (Procaccini et al., 2013; Maksimovic et al., 2014b), the *Gria1-/-* mice showed robust hyperlocomotion as compared to the WT mice, when placed into an unfamiliar environment (Figure 1A,B, ANOVA for genotype effect, *F*_1.49_ = 33.09, *P* < 0.001). They gradually habituated over the observation period of 2 h (time effect *F*_23,1127_ = 56.89, *P <* 0.001), although not down to the average level of WT mice (time × genotype interaction *F*_23,1127_ = 2.23, *P <* 0.01), as determined by the comparison of the mean path length of the last three timebins (genotype effect, *F*_1,10_ = 6.48, *P* < 0.05). CBD treatment dose-dependently attenuated the novelty-induced hyperlocomotion of *Gria1-/-* mice (treatment effect, *F*_4.49_ = 4.16, *P <* 0.01; genotype effect, *F*_1.49_ = 33.09, *P <* 0.001), while the locomotor activity of WT mice remained unaltered (treatment × genotype interaction, *F*_4.49_ = 3.74, *P <* 0.01; Figure 1, Bonferroni post-test, vehicle vs. CBD, *P* > 0.05). *Post hoc* comparisons showed that all doses of CBD (15, 60, and 100 mg/kg), except for the lowest dose (5 mg/kg), brought the hyperactivity of *Gria1-/-* mice back to the level of WT mice (Figure 1C, locomotor activity analyzed from the beginning of the trial until 30 min, and Figure 1D, locomotor activity analyzed over the whole 2 h trial, Bonferroni post-test, *P* > 0.05 for the genotype difference within all doses, except for 5 mg/kg, when it was *P* < 0.001). The 5 mg/kg dose seemed to fail to rescue the hyperactivity of the *Gria1-/-* mice in the beginning of the trial, but to facilitate the habituation in the later stage of the trial (Figure 1A,B, time × genotype × treatment interaction *F*_92,1127_ = 1.85, *P <* 0.001).

### Effects of Acute Cannabidiol Treatment on c-Fos Expression

**FIGURE 2:**
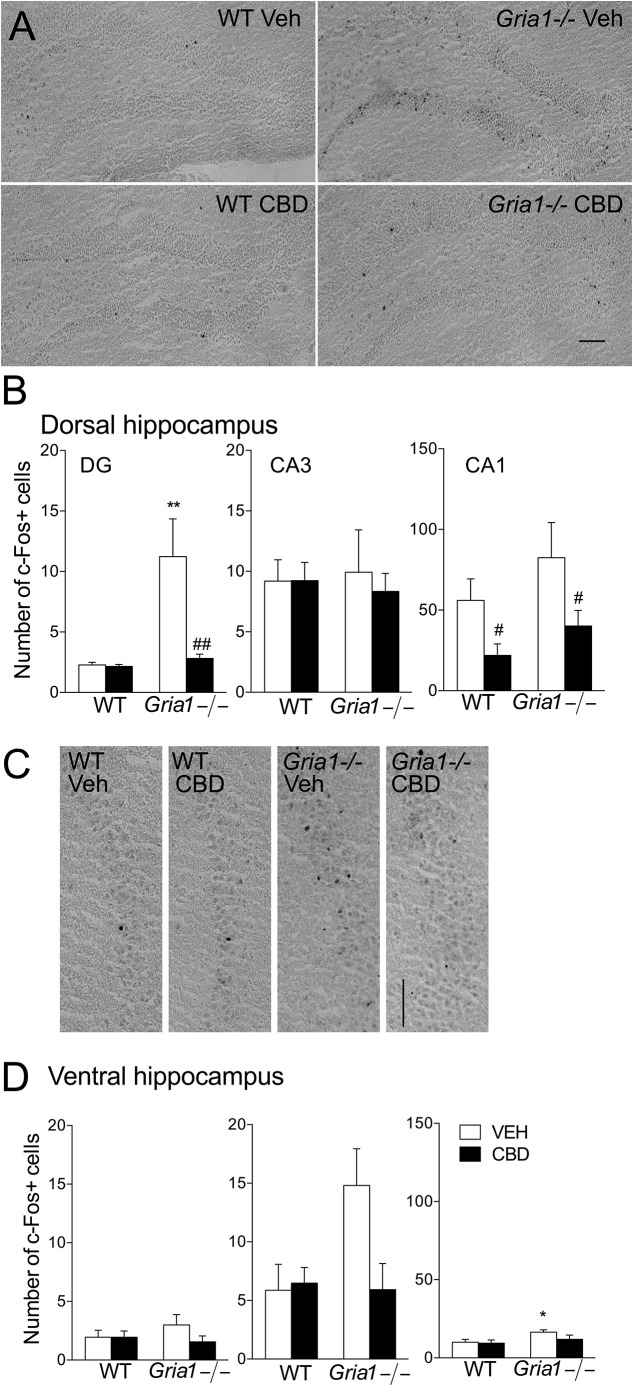
Expression of c-Fos protein in hippocampal subregions of the *Gria1-/-* and WT mice after a 2-h exposure to a novel environment with pre-treatment with vehicle or cannabidiol (15 mg/kg). **(A)** Representative images of the dorsal hippocampus. **(B)** Counts of c-Fos+ cells in the subregions of the dorsal hippocampus. Open bars for the vehicle- (VEH) treated and closed bars for cannabidiol- (CBD) treated groups. **(C)** Representative images of the ventral hippocampus. **(D)** Counts of c-Fos+ cells in the subregions of the ventral hippocampus. Means ± SEMs are shown for 4–6 mice per group. ^∗^*P* < 0.5, ^∗∗^*P* < 0.01 for the significances of the differences between genotypes after the same treatment, ^#^*P* < 0.05, ^##^*P* < 0.01 for the difference from the vehicle within the same genotype (Bonferroni post-test). Scale bar: 100 μm.

As the lowest effective dose of cannabidiol to block the novelty-induced hyperactivity in *Gria1-/-* mice was 15 mg/kg (Figure 1A,B), we analyzed the brain regional c-Fos protein expression after this dose (Figure 2). In the dentate gyrus granule cell layer of the dorsal hippocampus, the number of c-Fos-positive cells of the vehicle-treated *Gria1-/-* mice was significantly increased compared to the number of c-Fos positive cells in WT mice (Figure 2A, genotype effect, *F*_1,20_ = 9.32, *P <* 0.01). The CBD treatment of WT mice did not alter the number of c-Fos positive cells in the dentate gyrus, but CBD normalized the number of c-Fos positive cells in that of *Gria1-/-* mice (Figure 2B, treatment effect *F*_1,20_ = 7.34, *P <* 0.05; genotype × treatment interaction *F*_1,20_ = 6.90, *P <* 0.05). However, in the CA1 subfield, CBD treatment decreased c-Fos-positive cell counts in both *Gria1-/-* and WT mice (treatment effect, *F*_1,20_ = 7.35, *P <* 0.05). The c-Fos cell count in CA3 was equal between the genotypes (*F*_1,20_ = 0.001, *P >* 0.05) and not affected by the CBD treatment (*F*_1,20_ = 0.12, *P >* 0.05).

In the ventral hippocampus (Figure 2C,D), novelty-induced hyperactivity did not change the number of c-Fos positive cells in dentate gyrus subfield (genotype effect for DG, *F*_1,19_ = 0.24, *P >* 0.05). In the CA1 subfield, *Gria1-/-* mice had slightly higher number of c-Fos positive cells (genotype effect, *F*_1,19_ = 5.04, *P <* 0.05), but CBD treatment failed to reduce the cell count significantly (treatment effect *F*_1,19_ = 1.76, *P >* 0.05; genotype × treatment interaction *F*_1,19_ = 1.00, *P >* 0.05). In the CA3 subfield, novelty-induced hyperactivity did not change the number of c-Fos positive cells, although a non-significant trend was detected (genotype effect, *F*_1,19_ = 3.22, *P >* 0.05; genotype × treatment interaction, *F*_1,19_ = 4.13, *P* = 0.056).

The numbers of c-Fos-positive cells after treatment with CBD in extra-hippocampal brain regions are summarized in Supplementary Table S1. In the basolateral amygdala, CBD treatment reduced the number of c-Fos-positive cells in WT animals only (genotype × treatment interaction *F*_1,20_ = 4.74, *P <* 0.05). In the lateral septum, the number of c-Fos-positive cells increased in WT mice after CBD treatment (genotype × treatment interaction *F*_1,19_ = 8.54, *P <* 0.01). In the prelimbic cortex, the greater number of c-Fos-positive cells was observed in WT mice than in *Gria1-/-* mice, a difference that disappeared after CBD treatment (genotype × treatment interaction, *F*_1,20_ = 4.72, *P <* 0.05). Thus, compared to the hippocampus, extra-hippocampal brain areas of wild-type mice were hardly affected by the CBD. Only in the lateral septal nucleus of *Gria1-/-* mice the c-Fos response to CBD injection was opposite.

### Effect of Intrahippocampal Cannabidiol Treatment on Novelty-Induced Hyperactivity

**FIGURE 3:**
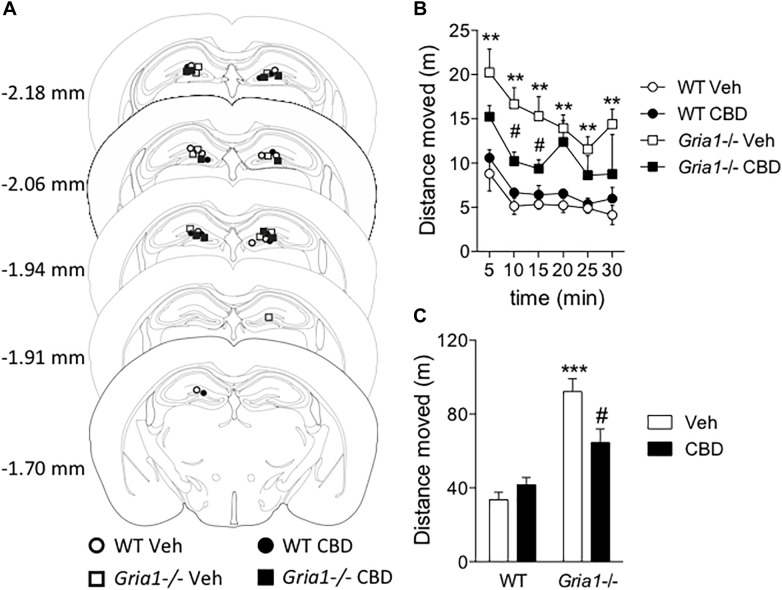
Intrahippocampal infusion of cannabidiol decreases novelty-induced hyperlocomotion of *Gria1-/-* mice. Animals were given an infusion with vehicle or cannabidiol (5 μg/side) followed by recording of distance moved for 30 min. **(A)** Injector tip placements within the dorsal hippocampus for all experimental groups. The brain images were modified with permission from Paxinos and Franklin (2001). **(B)** Distance traveled in a novel environment in 5-min periods. **(C)** Total distance traveled during 30-min trials. ^∗∗^*P* < 0.01 and ^∗∗∗^*P* < 0.001 for the significances of the differences between the genotypes after vehicle; ^#^*P* < 0.05 between vehicle and CBD within the same genotype (Bonferroni post-test). *N* = 10 for both genotypes. Veh, vehicle; CBD, cannabidiol.

The c-Fos-based neuronal activation mapping revealed the hippocampal complex as a main locus of novelty-induced hyperactivity in *Gria1-/-* mice (Figure 2 and Procaccini et al., 2011). Because systemic CBD both decreased the novelty-induced hyperactivity and re-adjusted the c-Fos positive cell score in the hippocampal dentate gyrus of *Gria1-/-* mice (Figure 1, 2), we sought to determine whether infusion of CBD into the dorsal hippocampus is sufficient to down-regulate the hyperactivity. We found that the intrahippocampal CBD microinjection (Figure 3A) significantly decreased, but did not completely reverse, the novelty-induced hyperactivity of *Gria1-/-* mice (Figure 3B,C), while not changing locomotor activity of WT mice (genotype effect, *F*_1,16_ = 49.08, *P* < 0.001; genotype × treatment interaction, *F*_1,16_ = 9.39, *P* < 0.01), supporting our hypothesis that the attenuation of the hyperactivity by CBD of *Gria1-/-* mice is mainly mediated by its action in the hippocampus.

### Chemogenetic Inhibition of Novelty-Induced Hyperactivity

**FIGURE 4:**
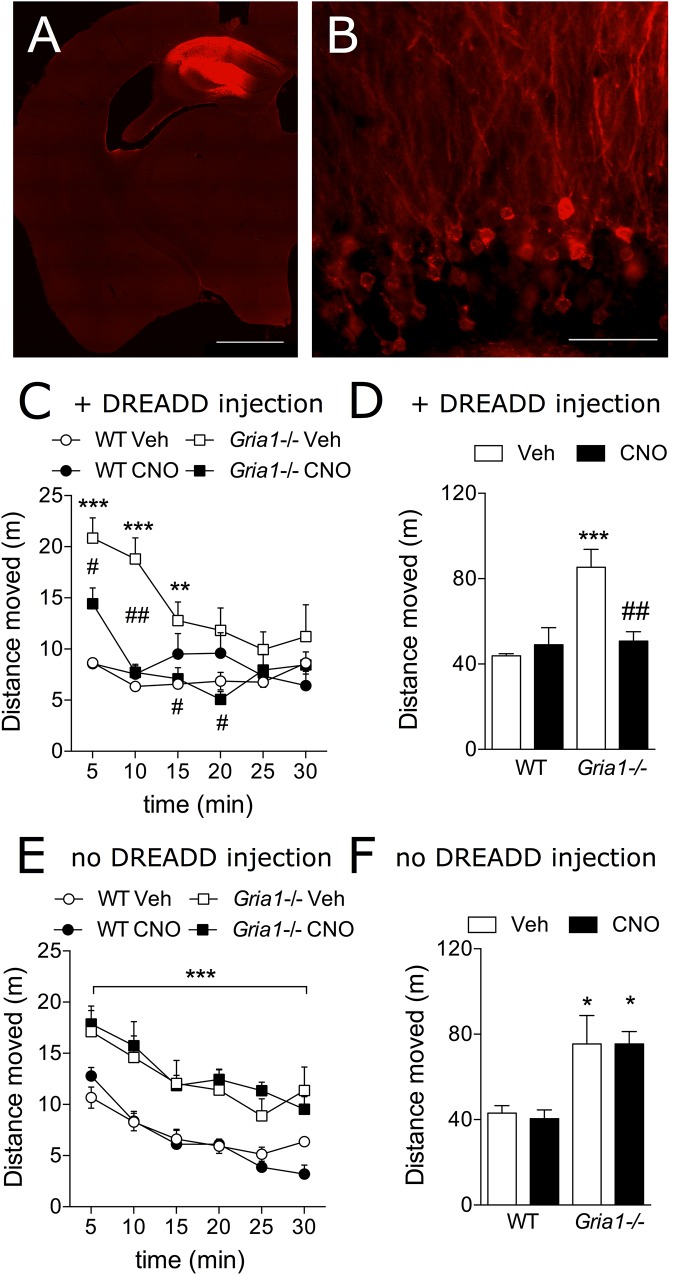
Chemogenetic inhibition of novelty-induced hyperlocomotion in *Gria1-/-* mice. **(A)** Virus injection resulted in hM4Gi-mCherry expression in the dorsal hippocampus. Hb, habenula; fi, fimbria; scale bar: 1 mm. **(B)** A close-up photograph displaying transduced neurons and neurites in the dentate gyrus. No transduced neurites were observed outside the hippocampus (not shown). Gr, granule cell layer; Mol, molecular layer; scale bar: 50 μm. **(C)** Distance traveled in a novel environment in 5-min periods in mice transduced with DREADDs. **(D)** Total distance traveled during the 30-min trial in mice transduced with DREADDs. ^∗∗^*P* < 0.01 and ^∗∗∗^*P* < 0.001 for the significances of the differences between the genotypes after vehicle; ^#^*P* < 0.05 and ^##^*P* < 0.01 between vehicle and CNO treatments within the same genotype (Bonferroni post-test), *n* = 10 for both genotypes. **(E)** Distance traveled in a novel environment in 5-min periods in non-DREADD expressing WT and *Gria1-/-* control mice. ^∗∗∗^*P* < 0.001 for the significances within drug treatment and between genotypes (ANOVA). **(F)** Total distance traveled during the 30-min trial in non-DREADD-expressing control mice. ^∗^*P* < 0.05 between genotypes and within drug treatment (Bonferroni post-test). *n* = 11–14. Veh, vehicle; CNO, clozapine-*N*-oxide.

The c-Fos-based neuronal activation mapping has revealed the hippocampal complex as a main locus of novelty-induced overactivity in *Gria1-/-* mice (Figure 2 and Procaccini et al., 2011). We used the clozapine-*N*-oxide (CNO) activation of the Gi-DREADD receptors (hM4Gi; Armbruster et al., 2007) to inhibit neuronal activity in the dorsal hippocampus of *Gria1-/-* mice and to reveal the anatomical and cellular substrate for the hyperlocomotion phenotype of the *Gria1-/-* mice (Figure 4A,B). *Gria1-/-* mice that received these bilateral AAV5-CaMKIIα-hM4Gi-mCherry injections in the dorsal hippocampus, CNO-induced DREADD inhibition of hippocampal CaMKIIα-expressing, excitatory cells decreased hyperlocomotion of *Gria1-/-* mice to the level of WT littermates (Figure 4C,D). In the WTs, CNO-induced DREADD inhibition in the same cell population had no modifying effect on the locomotor activity (treatment effect, *F*_1,16_ = 5.61, *P* < 0.05; genotype effect, *F*_1,16_ = 12.11, *P* < 0.01; genotype × treatment interaction, *F*_1,16_ = 10.27, *P* < 0.01). The effect persisted until the *Gria1-/-* mice habituated to the locomotor activity level of the WT mice (Figure 4C, time effect, *F*_5,80_ = 9.63, *P* < 0.001; time × genotype interaction, *F*_5,80_ = 7.46, *P* < 0.001; time × genotype × treatment interaction, *F*_5,80_ = 3.26, *P* < 0.01). In contrast, in DREADD-non-expressing mice, the drug CNO did not change the locomotor activity of either genotype (treatment effect, *F*_1,17_ = 0.47, *P* > 0.05; treatment × genotype interaction, *F*_1,17_ = 0.49, *P* > 0.05) and the *Gria1-/-* mice displayed hyperlocomotion (genotype effect, *F*_1,17_ = 15.61, *P* < 0.001) irrespective of the CNO treatment (Figure 4E,F).

## Discussion

Increased novelty-induced hyperactivity has been the most consistent and robust behavioral phenotypic alteration in mice with deficient AMPA-type glutamate receptor GluA1 subunits (for review see, Sanderson et al., 2008). In the present study, *Gria1-/-* mice again reproduced the hyperactive phenotype, also after stresses of the surgeries and intracerebral infusions, indicating that the model is stable for these kinds of experimental scrutiny.

The hippocampus is considered a most important brain area for spatial learning and adaptation (Bannerman et al., 2014; Moser et al., 2017). Inhibition of hippocampal principal neurons by DREADD receptors after CNO strongly blocked the novelty-induced hyperactivity in *Gria1-/-* mice, without affecting the locomotor activity of wild-type mice, indicating that the hippocampus is abnormally activated in the mutants during extensive exploration of novel environments. Such an increased hippocampal activity of *Gria1-/-* mice can be visualized by increased numbers of c-Fos expressing cells during exposure to unfamiliar surroundings [the present study (Procaccini et al., 2011)]. Both acute and chronic drug treatments have reduced both hippocampal c-Fos expression and behavioral hyperactivity (Procaccini et al., 2013; Maksimovic et al., 2014a,b).

Our data, using hippocampus specific Gi-DREADD and hippocampal infusion of CBD indicate that the hippocampal dysfunction is responsible for the impaired habituation of GluA1 deficient mice to new environments or situations. This view finds support by an inducible hippocampal deletion of GluA1 subunits from principal neurons during late adolescence which phenocopied the hyperactivity as seen in mice with global reduction of GluA1 (Inta et al., 2014). Since the DREADD system targeted mainly the dentate gyrus granule cells but was detected also in *cornu ammonis* regions (Figure 4A), it is likely that the DREADD-driven inhibition recruited more extensively the hippocampal formation leading to complete normalization of the novelty-induced hyperactivity in *Gria1-/-* mice. Similarly, the restoration of GluA1 in the hippocampus had attenuates the open field hyperactivity of *Gria1-/-* mice (Freudenberg et al., 2016). In our critical control experiment (MacLaren et al., 2016), the drug used to activate the DREADD receptors, clozapine-*N*-oxide (CNO) at the dose of 3 mg/kg, did not alter locomotor activities of the mouse lines without the DREADD expression (Figure 4E,F). This result is in line with the recent report that showed only higher than 5 mg/kg doses of CNO having partial clozapine-like effects in mice (Manvich et al., 2018).

Although hyperactivity and responses to novelty are often linked to increased dopaminergic mechanisms (Boekhoudt et al., 2016) and the *Gria1-/-* mice have been suggested to show hyperdopaminergic phenotype (Wiedholz et al., 2008), our previous experiments failed to find differential c-Fos expression between the *Gria1-/-* and WTs in the midbrain and striatal dopaminergic regions after exposure to a novel environment (Procaccini et al., 2011) and the hyperactive, hippocampally restricted GluA1 subunit-deficient mice had normal striatal dopamine levels (Inta et al., 2014). However, pharmacological antagonism of dopamine D2 receptors has been shown to non-selectively reduce hyperactivity in both *Gria1-/-* and WT mice (Wiedholz et al., 2008), making it difficult to fully exclude the role of putatively enhanced dopamine mechanisms in the *Gria1-/-* mice. Furthermore, recent neuronal pathway modulation on regulation of habituation and novelty preference in mice has indicated important interactive roles of the medial habenula and interpeduncular nucleus with the VTA, with the habenula activating the interpeduncular nucleus to recognize familiarity and the VTA inhibiting the interpeduncular nucleus to promote novelty seeking (Molas et al., 2017).

Despite that the dopaminergic dysfunction has a recognized role in schizoaffective disorders and might play a role in *Gria1-/-* mice, there is evidence rather pointing to indirect effects of cannabinoids on dopamine cell firing, by altering glutamate neurotransmission (Colizzi et al., 2016). The *Gria1-/-* mouse model has been sensitive to drugs that downregulate glutamate neurotransmission including several antiepileptic drugs and AMPA antagonists (Procaccini et al., 2013; Maksimovic et al., 2014a,b). CBD shows little target selectivity and has multiple actions on various receptors and ion channels (Izzo et al., 2009; Ibeas Bih et al., 2015). While our data on attenuation of locomotor hyperactivity by silencing glutamatergic neurons via G_i_-coupled DREADDs could imply the involvement of the inhibitory CB_1_ receptors, the inhibition of the presynaptic glutamate release via CBD action on the cannabinoid receptors have been largely dismissed (Ibeas Bih et al., 2015). One of the most interesting and relevant CBD actions is the inhibition of the G protein-coupled receptor 55 (GPR55). Hippocampally expressed G-protein-coupled receptor 55 (GPR55) induces Ca^2+^ release from presynaptic stores leading to increased synaptic neurotransmitter release (Sylantyev et al., 2013; Hurst et al., 2017). As GPR55 colocalizes presynaptically with the vesicular glutamate transporter 1 (Sylantyev et al., 2013), glutamate is the likely neurotransmitter for the GPR55-induced increase of excitatory transmitter release. Importantly, CBD inhibits GPR55-mediated excitatory drive in hippocampal synapses (Sylantyev et al., 2013), which may suggest a pharmacological significance in damping overactivated network within the hippocampal circuits (Kaplan et al., 2017). While a direct measurement of glutamate release in response to novelty-induced hyperactivity in *Gria1-/-* mice needs to be established, previous data suggest a role for glutamate release in novel environments (Bianchi et al., 2003). Therefore, the GPR55-regulated glutamate release provides a possible mechanism for the CBD-mediated downregulation of hippocampal excitatory activity and behavioral hyperactivity in *Gria1-/-* mice.

In addition to the activation of GPR55, CBD appears also to possess agonistic properties at serotonergic G_i_-coupled 5-HT_1A_ receptors, through which it can induce anxiolytic-like effects and mediate adaptation to stress (Joca et al., 2003; Russo et al., 2005; Rock et al., 2012). These receptors are localized at presynaptic terminals of glutamatergic synapses and their activation suppresses glutamatergic signaling (Cai et al., 2002). Thus, CBD actions on 5-HT_1A_ receptors might have contributed to the decrease in hippocampal neuronal overactivation in *Gria1-/-* mice.

The intrahippocampal CBD injection partially rescued the novelty-induced hyperactive phenotype in the *Gria1-/-* mice. Similar doses as in our study (bilaterally 5 μg/side) have been previously injected into the brain parenchyma, although not into the hippocampus. A 10-fold lower dose of CBD (0.4 μg/side) facilitated fear extinction, when injected bilaterally to rat infralimbic cortex (Do Monte et al., 2013). A higher unilateral periaqueductal CBD dose of 10 μg was required to produce an anxiolytic-like effect in rats, while 20 μg dose failed to affect the behavioral output (Campos and Guimaraes, 2008). In a summary, these studies suggest that bilateral 5 μg/side dose of CBD reaches appropriate concentration intrahippocampally to have an effect on various hippocampally expressed CBD targets, although the affinity of CBD to them might vary (Ibeas Bih et al., 2015).

Overall, our data suggest that silencing hippocampal glutamatergic neurons and systemic and intrahippocampal pharmacological treatment with CBD alleviate behavioral hyperactivity in *Gria1-/-* mice. CBD in this study has reproduced the effects of glutamate-modulating drug-treatments in blunting the excessive hyperlocomotion and the hyperactivity of the dorsal hippocampus in *Gria1-/-* mice (Procaccini et al., 2013; Maksimovic et al., 2014a,b). Although the precise brain circuitry and pharmacological targets involved in the CBD effect require further elucidation, our data contribute to the possibility that CBD actions on glutamatergic transmission in the hippocampus could be therapeutically applied to dampen hyperexcitable hippocampal and other brain circuitries.

## Data Availability

All datasets generated for this study are included in the manuscript and/or the Supplementary Files.

## Author Contributions

TA-a, MM, and ERK originated the study and wrote the first draft of the manuscript. TA-a, MM, and KD performed the experiments and statistical analyses. RS provided some mouse cohorts. All authors contributed to manuscript revision, read and approved the submitted version.

## Conflict of Interest Statement

The authors declare that the research was conducted in the absence of any commercial or financial relationships that could be construed as a potential conflict of interest.
