# Supplementary material for: Attenuation of Novelty-Induced Hyperactivity of Gria1-/- Mice by Cannabidiol and Hippocampal Inhibitory Chemogenetics
Source: Front Pharmacol. 2019 Mar 29;10:309. doi: 10.3389/fphar.2019.00309 (PMC6449460; doi:10.3389/fphar.2019.00309)
Supplement: Supplementary file 1 [file Data_Sheet_1.PDF]

Table S1

Effect of acute treatment with systemic cannabidiol on the number of c-Fos protein-positive cells/0.1 mm^2 in different brain regions of *Gria1−/−* and WT animals.

Brain region	Genotype	Treatment		Two-way ANOVA
		Vehicle	Cannabidiol	
Amygdala, basolateral nucleus	WT	4.8±0.2	3.4±0.5 $^{\#}$	G×T
	Gria1−/−	3.9±0.5	4.4±0.5	
Amygdala, central nucleus	WT	1.5±0.3	1.8±0.3	
	Gria1−/−	1.1±0.5	2.0±0.5	
Lateral septal nucleus, ventral part	WT	12.0±2.9	19.0 ±2.6 $^{\#}$	G×T
	Gria1−/−	17.±2.0	10.9±1.1 *	
Prelimbic cortex	WT	2.8±0.5	1.3±0.4 $^{\#\#}$	G×T
	Gria1−/−	1.4±0.3 *	1.4±0.3	
Caudate-putamen	WT	0.3±0.2	0.3±0.2	
	Gria1−/−	0.9±0.3	0.7±0.3	
Nucleus accumbens, core	WT	0.6±0.4	0.2±0.1	
	Gria1−/−	0.3±0.2	0.2±0.1	

The data are presented as mean ± SEM (n = 4-6; few regions were lost during sectioning). Letters show significance of factors after two-way ANOVA (G for genotype, T for treatment, and G×T for interaction between genotype and treatment). Bonferroni post-hoc test: * *P*< 0.05, for the difference between genotypes after the same treatment, $^{\#}$ *P*< 0.05, $^{\#\#}$ *P*< 0.01, for the difference from the vehicle within the same genotype.
